# Quantum vaccinomics platforms to advance in vaccinology

**DOI:** 10.3389/fimmu.2023.1172734

**Published:** 2023-06-15

**Authors:** José de la Fuente, Marinela Contreras

**Affiliations:** ^1^ SaBio. Instituto de Investigación en Recursos Cinegéticos IREC-CSIC-UCLM-JCCM, Ciudad Real, Spain; ^2^ Department of Veterinary Pathobiology, Center for Veterinary Health Sciences, Oklahoma State University, Stillwater, OK, United States

**Keywords:** vaccine, vaccinomics, vaccinology, immunology, quantum

## Abstract

The opinion flows from Introduction to the immunological quantum that requires a historical perspective, to Quantum vaccine algorithms supported by a bibliometric analysis, to Quantum vaccinomics describing from our perspective the different vaccinomics and quantum vaccinomics algorithms. Finally, in the Discussion and conclusions we propose novel platforms and algorithms developed to further advance on quantum vaccinomics. In the paper we refer to protective epitopes or immunological quantum for the design of candidate vaccine antigens, which may elicit a protective response through both cellular and antibody mediated mechanisms of the host immune system. Vaccines are key interventions for the prevention and control of infectious diseases affecting humans and animals worldwide. Biophysics led to quantum biology and quantum immunology reflecting quantum dynamics within living systems and their evolution. In analogy to quantum of light, immune protective epitopes were proposed as the immunological quantum. Multiple quantum vaccine algorithms were developed based on omics and other technologies. Quantum vaccinomics is the methodological approach with different platforms used for the identification and combination of immunological quantum for vaccine development. Current quantum vaccinomics platforms include *in vitro*, *in music* and *in silico* algorithms and top trends in biotechnology for the identification, characterization and combination of candidate protective epitopes. These platforms have been applied to different infectious diseases and in the future should target prevalent and emerging infectious diseases with novel algorithms.

## Introduction to the immunological quantum

Vaccines are one of the most important achievements in human history. From classical 3Is (isolate-inactivate-inject) to recombinant vaccines and recent vaccinomics approaches, vaccines have prevented millions of deaths worldwide (1–4). However, development of effective vaccines against infectious diseases such as tuberculosis, acquired immunodeficiency syndrome (AIDS), malaria, Lyme disease or Crimean-Congo Hemorrhagic Fever (CCHF) causing millions of deaths annually is still a challenge.

Quantum biology was proposed by Pascual Jordan in 1932 based on the dynamics of biological living systems to maintain the non-equilibrium state (4, 5). Then and based on the double helix structure of DNA, proton tunneling was proposed in 1963 as the quantum mechanics mechanism of DNA point mutations (6). Additionally, stochastic models have been proposed for gene regulation based on gene activation and inactivation by random association and dissociation events (7). These findings support the concept of quantum immunology based on the random processes of electronic structure of molecular interactions behind peptide immunogenicity present in the immune system (4, 8). Then, in allusion to Albert Einstein’s definition of the proton as a quantum of light, immune protective epitopes were proposed as the immunological quantum (9).

## Quantum vaccine algorithms

Using a bibliometric analysis by searching “quantum + vaccine + algorithm” in PubMed (https://pubmed.ncbi.nlm.nih.gov; January 16, 2023), the results provided 14 references. The recent origin of these algorithms starts with advances in deep sequencing and structural studies applied to protective candidate antigens to develop safer and more efficacious vaccines (10). Reverse vaccinology approaches such as Vacceed were developed for *in silico* vaccine candidate discovery (11). Algorithms and methods based on amphipathicity profiles of proteins, sequence motifs, quantitative matrices (QM), artificial neural networks (ANN), support vector machines (SVM), quantitative structure activity relationship (QSAR), T-cell major histocompatibility complex (MHC) class I binding prediction and molecular docking simulations among others were developed to predict T-cell epitopes (12, 13). Quantum chemical calculations to predict biological function were applied to T-cell receptor interaction with a peptide/MHC class I (14). Recently, an algorithm was proposed using semi-empirical quantum mechanical methods for calculating peptide-MHC class I and II molecules binding energy for the rational design of T-cell epitopes with application in vaccinology (15).

Mathematical combinatorial and computational techniques for drug discovery such as topology, combinatorics, graph theory and knot theory could be also applied to vaccinology (16). For example, facing recent challenges associated with severe coronavirus disease (COVID-19), the interactions between SARS-CoV-2 spike glycoprotein (S protein) and human angiotensin-converting enzyme 2 (hACE2) were characterized to identify potential vaccine candidates. Machine learning algorithms were applied to identify changes in infrared spectra associated with variations of the secondary structure of S protein for developing faster than conventional quantum chemistry calculations of real-time spectroscopy of protein dynamics (17). Combining this approach with antibody isotype S epitope mapping in different patient cohorts (18) may facilitate the identification of candidate protective epitopes for vaccine development.

Computational protein design algorithms are important for the combination of immunological quantum in vaccine antigens and have the potential to increase the accuracy and reliability of vaccine chimeric antigens (19). An innovative approach using biocompatible, near-infrared quantum dots (QDs) was recently proposed for the delivery of intradermal QDs with reliable code information together with vaccines for developing tools for vaccine decentralized data storage and biosensing (20).

## Quantum vaccinomics

**Figure 1:**
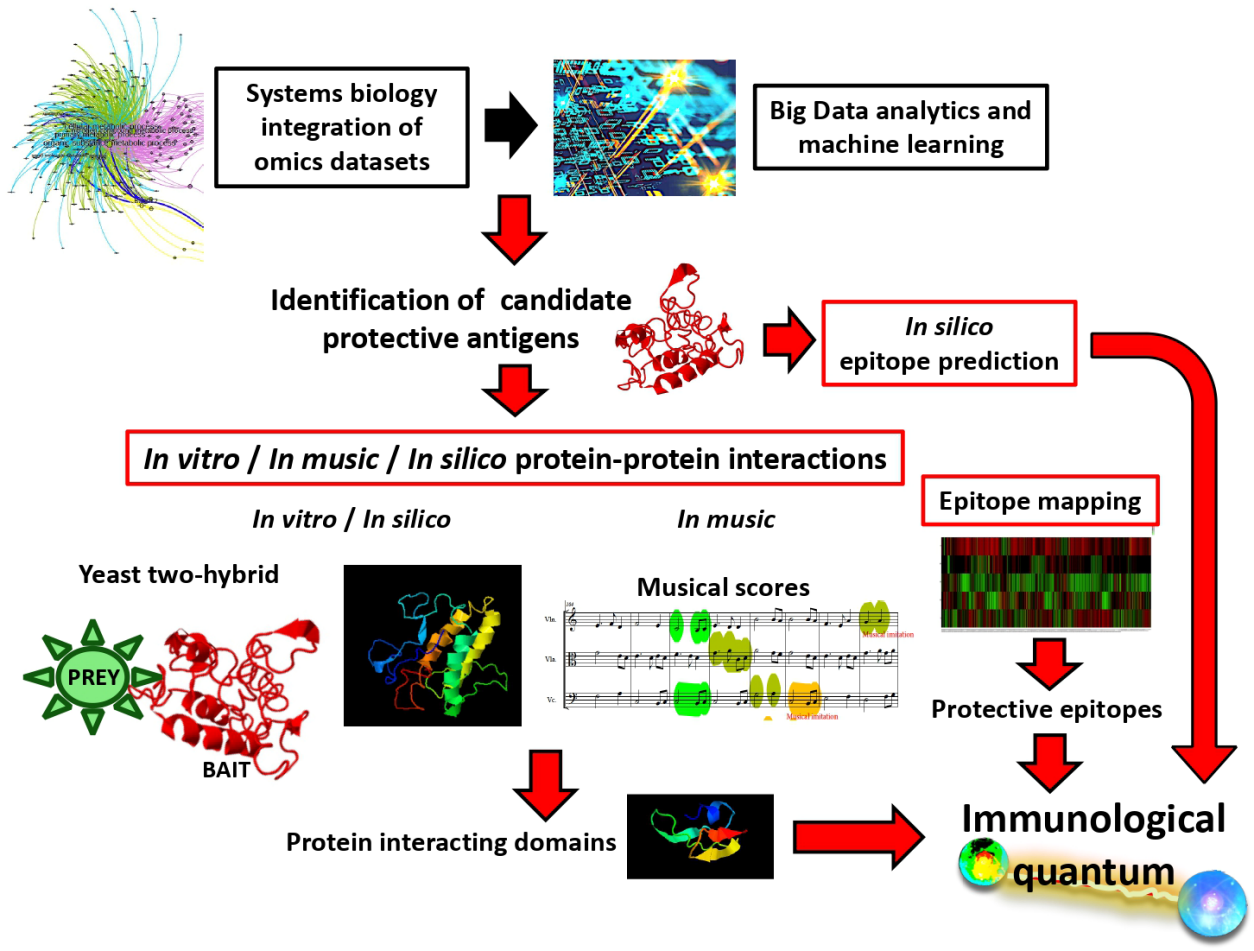
Quantum vaccinomics platforms for the identification of immunological quantum and design of vaccine chimeric protective antigens. Candidate protective antigens are identified using systems biology integration of omics dataset combined Big Data analytics and machine learning. Then, immunological quantum can be identified using *in silico* algorithms or epitope mapping for predicting protective epitopes or using *in vitro*, *in silico* and *in music* approaches for the identification of domains involved in protein-protein interactions.

Vaccinomics was defined as the application of immunogenetics and genomics to study the molecular mechanisms in response to vaccines (21). In the vaccinomics platform, systems biology integration of omics datasets combined with Big Data analytics and machine learning allow the identification of candidate vaccine protective antigens (2, 4, 22–28) (**Figure 1**). Quantum vaccinomics was then proposed as the methodological approach with different platforms for the identification and combination of immunological quantum for vaccine development (4, 29–33). Quantum vaccinomics platforms include *in silico* prediction and epitope mapping of immunological quantum as well as *in vitro*, *in music* and *in silico* characterization of protein-protein interactions to identify protein interacting domains as candidate protective epitopes (29–33) (**Figure 1**). These platforms facilitate antigen combination and including probiotics and post-translational modifications such as glycan alpha-gal to boost protective immune response to vaccination (34, 35). In this way, quantum vaccinomics platforms include top trends in biotechnology such as Big Data, gene sequencing and editing, precision medicine, bio manufacturing, and synthetic biology.

As recently reported (33), vaccines may elicit protective response in some species but in others prevalent antibodies may recognize non-protective epitopes. Using sera from both vaccinated protected and non-protected species it is possible to conduct a microarray epitope mapping to identify immunological quantum for the design of a chimeric protective antigen (33). Epitope mapping can also be used to identify immunological quantum recognized by immunized hosts and reactive to different pathogen and vector species. For example, using sera from tick Subolesin-immunized cattle it was possible to identify B-cell reactive epitopes in different tick species (32). This information could then be used to combine protective epitopes and design a vaccine chimeric antigen with improve protection against multiple tick species parasitizing on the same host.

The collaboration between science and art has shown an impact on approaching scientific and social challenges (e.g., (29, 36–38)). In this context, musical algorithms to translate gene/protein sequences into music provide insights into biomolecule evolution and interactome (30, 39). Protein interactome involves functional sequences that are not highly exposed to the immune system but play a key function and thus constitute candidate protective epitopes. The information obtained from *in music* approaches translates into the identification of protein-protein interacting motifs that in combination with *in vitro* (e.g., yeast two-hybrid) and *in silico* methods provide candidate immunological quantum for vaccine antigen design (29, 30) (**Figure 1**). *In silico* computational algorithms applied to vaccine discovery transforms digital abstractions of this complex interdisciplinary and interdependent system into candidate protective antigens (31, 40, 41).

## Discussion and conclusions

Despite the advances represented by quantum vaccine algorithms, limitations of these algorithms were approached using quantum vaccinomics. For example, Van Regenmortel (42, 43) discussed that peptide antigenicity can be chemically and structurally modified to improve antibody-peptide interactions, but it does not necessarily improve immunogenicity mediated by multiple factors of the host immune system. Using human immunodeficiency virus (HIV) model, Van Regenmortel (44) illustrates the limitations of reductionist methods, systems biology and structure-based reverse vaccinology to address the complexity of the human immune system for a rational design of anti-HIV vaccines for the prevention of acquired immunodeficiency syndrome (AIDS). However, quantum vaccinomics combines different platforms including not only *in vitro*, *in music* and *in silico* characterization of protein-protein interactions but also mapping of B-cell reactive protective epitopes and characterization of cellular immune mechanisms associated with protection in response to vaccine using integration of omics datasets (18, 29, 32, 33). For example, quantum vaccinomics was successfully applied to the tick protective antigen Subolesin. Subolesin was discovered by expression library immunization in the mouse model of *Ixodes ricinus* tick infestations (45). Protective linear B-cell and conformational epitopes were mapped and *in silico* modeling of protein structure was then used to identify and combine candidate protective epitopes in the chimeric antigen Q38 (31, 46). The Q38 antigen was validated using other quantum vaccinomics algorithms (29, 32) and elicited a protective immune response against tick infestations (47).

The combination of the different quantum vaccine and quantum vaccinomics approaches is important to provide reliable information on the proposed immunological quantum. These results need to be validated *ex vivo* and *in vivo* to advance in vaccine development. Our research in this area is mainly focused on ticks and tick-borne diseases (29–33), but quantum vaccinomics approaches have been applied to other diseases such as COVID-19 (17), tuberculosis (48), AIDS (41) and neosporosis (40). Future directions using quantum vaccinomics approaches should target highly prevalent and emerging infectious diseases.

Novel platforms and algorithms will be developed to further advance in quantum vaccinomics for the development of vaccines and other control interventions. These novel platforms include (a) use of commensal bacteria to produce and secrete protective antigens to interfere with pathogen infection or serve for vaccine delivery (49), (b) combination of vaccines with probiotics (e.g., with high alpha-gal content) and heat inactivated mycobacteria to serve as adjuvants/immunostimulants (50, 51), (c) new vaccine delivery platforms (e.g., nanoparticle (NP)-based formulations, lipid NP-mRNA, viral vectors, virus-like particles) to stimulate innate and trained immunity and boost protective immune response (52), (d) oral vaccine formulations to improve safety and access to developing countries (50), and (e) stimulating trained immunity mechanisms in response to vaccination (51).

## Author contributions

Both authors listed have made substantial, direct and intellectual contributions to the work and approved it for publication.
